# Long-Term Unemployment and Suicide: A Systematic Review and Meta-Analysis

**DOI:** 10.1371/journal.pone.0051333

**Published:** 2013-01-16

**Authors:** Allison Milner, Andrew Page, Anthony D. LaMontagne

**Affiliations:** 1 The McCaughey Centre: VicHealth Centre for the Promotion of Mental Health & Community Wellbeing, Melbourne School of Population Health, The University of Melbourne, Melbourne, Victoria, Australia; 2 Discipline of Epidemiology and Biostatistics, School of Population Health, the University of Queensland, Brisbane, Queensland, Australia; Tehran University of Medical Sciences, Iran (Islamic Republic of)

## Abstract

**Purpose:**

There have been a number of reviews on the association+ between unemployment and suicide, but none have investigated how this relationship is influenced by duration of unemployment.

**Method:**

A systematic review and meta-analysis was conducted of those studies that assessed duration of unemployment as a risk factor for suicide. Studies considered as eligible for inclusion were population-based cohort or case-control designs; population-based ecological designs, or hospital based clinical cohort or case-control designs published in the year 1980 or later.

**Results:**

The review identified 16 eligible studies, out of a possible 10,358 articles resulting from a search of four databases: PubMed, Web of Knowledge, Scopus and Proquest. While all 16 studies measured unemployment duration in different ways, a common finding was that longer duration of unemployment was related to greater risk of suicide and suicide attempt. A random effects meta-analysis on a subsample of six cohort studies indicated that the pooled relative risk of suicide in relation to average follow-up time after unemployment was 1.70 (95% CI 1.22 to 2.18). However, results also suggested a possible habituation effect to unemployment over time, with the greatest risk of suicide occurring within five years of unemployment compared to the employed population (RR = 2.50, 95% CI 1.83 to 3.17). Relative risk appeared to decline in studies of those unemployed between 12 and 16 years compared to those currently employed (RR = 1.21, 95% CI 1.10 to 1.33).

**Conclusion:**

Findings suggest that long-term unemployment is associated with greater incidence of suicide. Results of the meta-analysis suggest that risk is greatest in the first five years, and persists at a lower but elevated level up to 16 years after unemployment. These findings are limited by the paucity of data on this topic.

## Introduction

Past review studies have provided strong evidence of the relationship between unemployment and suicide [1]–[4]. However, the magnitude of the relative and attributable risk associated with unemployment has been contested, as various authors have suggested that the effect of socio-economic factors on suicide tend to be overestimated when the contribution of psychiatric disorders is not taken into account [5]. This assumption is problematic as it assumes that mental illness and unemployment have separate and independent effects on suicide when, in fact, mental illness is a likely intermediatory factor between unemployment and suicide [6].

A further problem is that many past studies view unemployment as a simple static and binary state (i.e. unemployed versus employed), thereby ignoring the dynamic nature of employment status. A person may only be without a job for a finite period before re-employment, or from unemployment they may exit the labour market. The importance of considering the potential risks associated with length of unemployment has recently been recognised in epidemiological studies, which have demonstrated that longer durations of unemployment affect cause-specific and all-cause mortality differentially over time [7], [8]. Variation in the effect of unemployment duration has also been shown in studies of psychological wellbeing, which have demonstrated pronounced adverse mental health consequences the longer a person is unemployed [9]. At the same time, past meta-analyses on mental health outcomes and all-cause mortality [7], [9] suggest a possible amelioration or “adjustment” to the adverse effects of unemployment on mental health and mortality over time.

There has been no systematic review and meta-analysis on length of unemployment and suicide. However, given the results of the meta-analyses [7], [9] discussed above, there is good reason to suspect that unemployment may have differential effects on suicide over time. This systematic review and meta-analysis seeks to summarise evidence to date on the effects of duration of unemployment on suicide attempts and deaths. The main hypotheses of the study were that: 1) long term unemployment would be a risk for suicide attempt and mortality, and; 2) the relationship between unemployment and suicide would vary over time.

## Methods

Unemployment duration was measured as either a continuous (e.g., number of days since the loss of a job) or categorical (e.g., short versus long term unemployment) variable. The study protocol was based on the Preferred Reporting Items for Systematic Reviews and Meta-Analyses (PRISMA) (http://www.prisma-statement.org/).

### Databases and search terms

The search was conducted using four databases: Pub Med, Web of Knowledge, Scopus and Proquest. These databases were chosen to ensure that the literature search strategy comprehensively examined research from medicine, epidemiology, sociology and psychology. A secondary search of reference lists was undertaken from within retrieved articles. Search terms used for the search were: suicid* OR self injur* OR deliberate self harm AND job loss OR unemploy*. Authors were contacted to identify additional statistical details on retrieved studies. The first author conducted the initial searches and shortlisting. Subsequent searches and checking was undertaken by the other two authors, with mismatches in classification resolved by consensus.

### Inclusion and exclusion criteria

Articles were considered if the search terms were included in the abstract or title of the article and were published in the last thirty years (i.e. 1980 or later), which was when the last review study on the unemployment and suicide was conducted (criterion A) [2]. After a review of the title and abstract, review articles, editorials and papers not in English were excluded. Only peer reviewed research was considered (criterion B). Duplicates were also removed. Following this, the abstract and text were reviewed to assess whether unemployment was a key independent variable of interest (criterion C) and suicide was a measured outcome variable (criterion D). Among the remaining articles, preference was given to those articles and scholarly pieces that measured the effect duration of unemployment over time (criterion E), or the risk associated with suicide following job loss (criterion F).

### Study design and variables of interest

This review included studies conducted at the individual and the aggregate (ecological) level. Qualitative and case-series studies were excluded. Studies were classified according to whether they were: (1) population-based cohort or case-control designs; (2) population-based ecological designs, or; (3) hospital based clinical cohort or case-control designs. Suicidal behaviours could be measured as either non-fatal (suicide attempts) or fatal (suicide).

### Data extraction

The data extracted from identified studies included suicide attempt or death, duration of unemployment or follow up from the point of unemployment, and results by sex (where available). The results of studies were described individually using summary measures such as risk or rate ratios.

### Meta- analysis

Of those studies included in the overall systematic review, only a subset of longitudinal cohort studies on mortality were considered eligible for meta-analysis. The reason for this was that these were the only studies that provided comparable measurements of exposure (unemployment duration or follow up from the time of unemployment) and outcome (suicide). Pooled effect size and 95% confidence intervals were calculated using random effects meta-analysis using the inverse variance (Dersimonian and Laird) method. The effects assessed to be eligible in in the meta-analysis included hazard, odds or rate ratios. Heterogeneity between studies was assessed using the *I* ^2^ statistic, which provided an estimate of the percentage of variability in the outcome that is due to differences in exposure-outcome association. Where possible, adjusted estimates (with 95% confidence intervals) were used and results were stratified by sex. The possibility that unemployment would have nonlinear effects over time was tested as an *a priori* hypothesis based on the findings of a previous meta-analysis [7], which demonstrated the most significant levels of relative risk of all cause-mortality occurred within either five years or five to ten years of unemployment, and was reduced thereafter. The meta-analysis was carried out in Stata Version 12 [10]. Meta-regression was conducted to assess the impact of duration of unemployment as a source of heterogeneity. Funnel plots were used to assess publication bias and small study effects [11].

## Results

**Figure 1:**
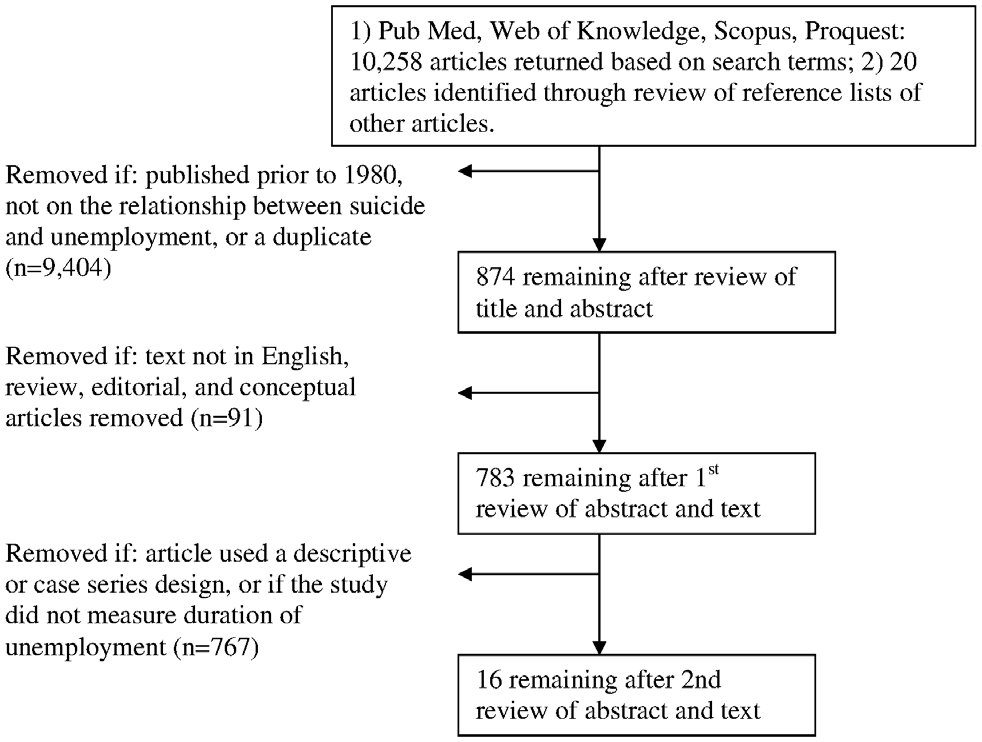
Search strategy following PRISMA guidelines.

The process of inclusion and exclusion of articles can be seen in Figure 1. After reviewing titles and abstracts, articles were excluded if they did not specifically investigate the association between unemployment and suicide, were duplicate studies or published prior to 1980. Among the 874 remaining articles, 87 editorial, review and conceptual articles were removed. Articles were also excluded if the article was not in English. The abstract and text were reviewed to exclude those articles that were descriptive case-series or qualitative designs. Over 300 articles were ecological studies, conducted either cross-sectionally or longitudinally, in which unemployment duration and/or suicide were not the primary variables of interest. Restricting studies to those where a measure of duration of unemployment or time since job loss was included resulted in 16 articles relevant to this systematic review (Table S1).

### Study quality

The quality of studies was assessed before inclusion in the systematic review and meta-analysis, based on published recommendations [12]. Seven studies of suicide used cohort designs [8], [13]–[18], while three examined the relationship between unemployment duration and suicide using an ecological design [19]–[21]. Four other studies examined unemployment duration in relation to suicide attempts using data from hospitals [22]–[25], while two studies examined suicide attempts and ideation using a case-control design [26], [27]. The source populations were more representative in large cohort studies than those in clinical cohorts based in smaller hospitals. This is likely related to differences in the ability to collect population-level data on suicide attempts versus mortality.

There were considerable differences in the measurement of unemployment duration between studies (exposure) (Table S1), with some studies defining unemployment as a categorical variable and others measuring it continuously. The representativeness of exposure was higher in population representative cohort studies than clinical cohort studies. Clinical cohort studies relying on self-report of unemployment status may be subject to non-differential selection bias.

Cohort studies included in the meta-analysis were not subject to this problem as unemployment data was usually ascertained from objective data sources (e.g., welfare payment or official employment figures). However, these sources may suffer from a lack of sensitivity and specificity in both the exposure and the outcome, and issue that was not generally addressed in the design of studies. This bias is likely to be non-differential, with an equal likelihood of affecting both cases and controls. Loss to follow-up is another issue that may affect longitudinal cohort studies. These limitations not withstanding, retrospective cohort designs capturing data at the population level were deemed to be the highest quality studies available.

### Attempted suicide

Four clinical cohort studies examined employment status in those persons attending a hospital after engaging in suicidal behaviours [22]–[25]. Among the earliest published were hospital-based studies by Platt and colleagues [22], [23], which found consistently higher rates of suicide among the long-term unemployed than employed or short-term unemployed suicide attempters (Table S1). There were two population-based cohort studies of attempted suicide in young people (under 25 years) in New Zealand [26], [27]. In comparison to those persons who experienced no unemployment, the relative risk of attempted suicide in those who had experienced ≤6 months of unemployment was 1.31 (95% CI 0.94 to 1.82), and in those who had experienced over 6 months was 1.72 (95% CI 0.89 to 3.32) after adjusting for mental disorders [27].

### Suicide

An ecological study on suicide over the period 1948–1978 in the USA [20], found that longer duration (measured as a continuous variable) was associated with higher male and female suicide rates. Another ecological study by Shah [19] found no relationship between long term unemployment (time period undefined) and suicide rates across 27 countries. The outcome variable in this study was suicide rates in those aged 65 years and over, a population not widely represented in the labour market.

Recently, Classen and Dunnn [21] conducted an ecological study on the relationships between unemployment duration associated with mass lay-off events and suicide across 50 states of the USA. Results of this study indicated that a rise in the number of workers who were unemployed between 15 and 26 weeks was associated with an increase in suicides among males, but this relationship was no longer apparent after 26 weeks. For females, unemployment longer than 5 weeks was associated with an increase in suicides.

### Meta-analysis

The remaining studies on suicide were retrospective cohort designs capturing data at the population level in Sweden, Finland, and Denmark [8], [13]–[18]. As discussed above, these studies generally were assessed to have substantially higher quality than the papers reviewed above. The data sources used in these studies originated from longitudinal employment and mortality registers. Four studies obtained information on psychiatric history from hospital databases [8], [14], [17], [18], while one other obtained psychiatric information from military conscription testing [15]. Following closer inspection of the data sources, one study [13] was excluded from the meta-analysis as its analysis of unemployment duration and suicide was conducted on a limited sample of cases (those who were previously admitted to a hospital for a psychiatric disorder).

**Figure 2:**
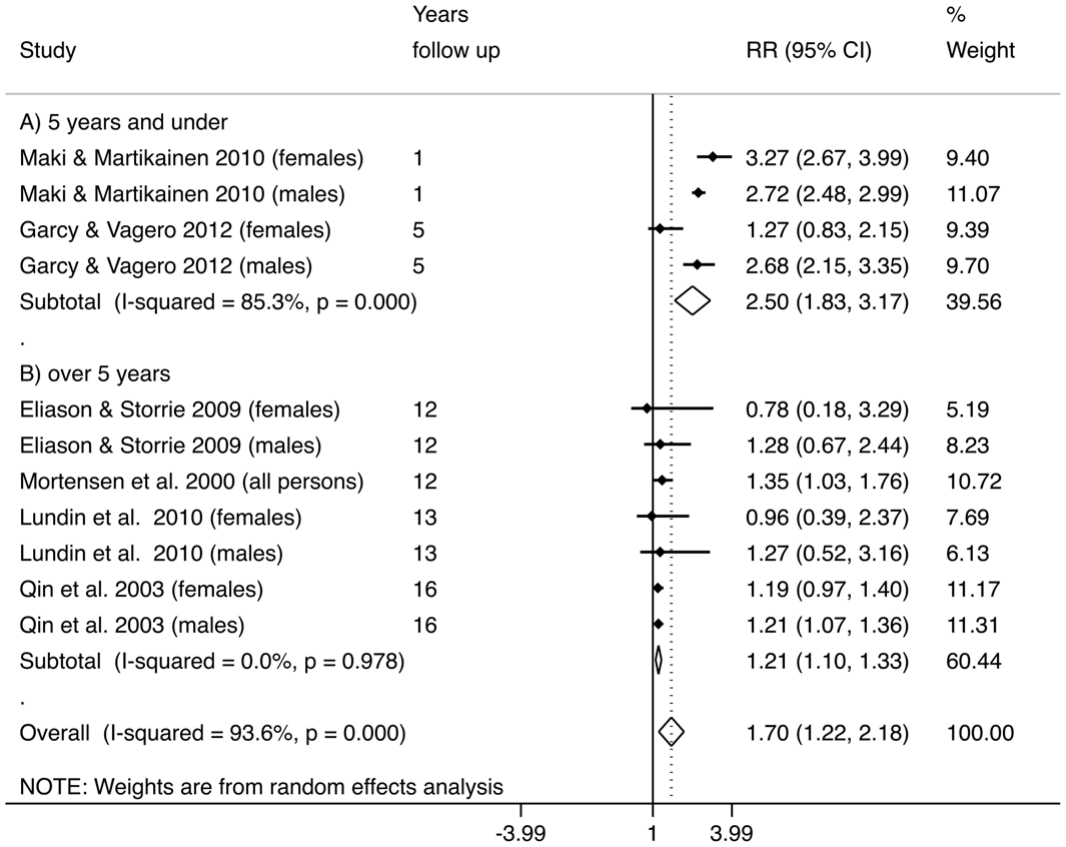
Forest plot of suicide risk following unemployment, follow up over time, results of meta-analysis. Reference is the employed population.

The majority of studies used hazard ratios as outcome measures, while one study used odds ratios. As suicide can be considered a rare outcome in a population, these measures of risk were seen as comparable [28]. As can be seen in Figure 2, the overall pooled relative risk of suicide associated with longer unemployment (average follow-up time 7.8 years) compared to those currently employed was 1.70 (95% CI 1.22 to 2.18). Inspection of the *I* ^2^ indicated a high degree of heterogeneity between studies (93.6%). This variation between studies and initial inspection of the forest plot (Figure 2) was consistent with the a priori hypothesis of a non-linear relationship between unemployment and suicide over time. Results were then stratified by studies with follow-up periods below and above five years after unemployment. The overall pooled relative risk in studies with follow up less than five years was 2.50 (95% CI 1.83 to 3.17) compared to those currently employed, while the risk in those studies with follow up periods between 12 and 16 years was 1.21 (95% CI 1.10 to 1.33) compared to those currently employed.

Meta-regression was used to assess the impact of duration of unemployment as a source of heterogeneity and revealed significant differences based on time to follow up. Studies with a longer duration of follow up had significantly lower RR than those that had a shorter follow-up period. Meta-regression was also used to assess the possible influence of SES and other factors such as mental illness. The results of this test suggested that controlling for these factors had no significant influence on results.

Possible publication bias and small study effects were assessed through inspection of a funnel plot (see Figure S1). This indicated an asymmetric plot, with smaller studies showing larger effect sizes, which may suggest publication bias. Following this, we investigated funnel plot asymmetry using Egger and colleagues' 1997 test for small study effects in meta-analysis [11]. The estimated bias coefficient was −1.86 with a standard error of 2.2, giving a p-value of 0.414. This test provides no evidence of small study effects, although the large standard error indicates considerable heterogeneity in results. Further, it is important to note that results of this test may be limited by the small number of studies included in analysis.

## Discussion

This study systematically reviewed the evidence on the relationship between unemployment duration and suicide. Unemployment duration was identified as the main study factor in 16 studies. Based on this relatively small number of studies (compared to the proportionally larger number of studies of general unemployment and suicide), it appears that the effect of unemployment duration is not frequently explored in suicide research. Even within those studies that examined differences in unemployment duration, this was often poorly assessed. For example, two ecological studies failed to explain how long a person was unemployed before they were classified as “long term”, [19], [20] and the majority of the cohort studies included in meta-analysis did not provide a clear indication of whether employment status was assessed continuously or periodically throughout the follow-up period.

There is tension in epidemiological research about whether the primary objectives of meta-analytic studies should be either the estimation of an overall summary or average effect across studies, or the identification and estimation of differences between studies [28]. In this study, considerable efforts have been made to illustrate the extent of differences between studies and to emphasise sources of heterogeneity. Despite these differences, consistent findings were still apparent across identified studies. Long term unemployed persons (compared to short-term unemployed persons) had a greater number of suicide attempts and were at increased risk of re-attempting suicide in both the clinical context and the general population. Findings from the meta-analysis also provide some evidence that longer durations of unemployment were associated with a higher relative risk of suicide compared to those currently employed and that the association is likely to be nonlinear with stronger relative risk estimates for earlier follow-up periods [8], [16] compared to later follow-up periods [14]–[17]. Further evidence is needed in order to understand when suicide risk peaks.

Psychological studies on unemployment suggest that there is a critical time period of between three months and a year during which people may be most at-risk of mental disorder [29], [30]. This time is thought to coincide with the growing sense of hopelessness that may accompany the perceived transition from short- to long-term unemployment [9], [29]. Some findings suggest that symptoms of mental ill health may stabilise at an elevated level during the second year of unemployment, before being associated with a renewed increase in distress in the long-term unemployed [9]. A recent meta-analysis of all–cause mortality also suggests that the influence of duration changes over time, with a higher risk of mortality at 5 years follow-up (to 73% increased risk of mortality) but reducing at 10 years follow-up to a level above that observed at baseline (to 42% increased risk of mortality) [7]. These findings are similar to the studies reported in this review, which suggests that there may be a reduction in risk after a period of 5- years of follow up.

The definition and measurement of unemployment duration was a problem in studies included in our meta-analysis, as most studies provided incomplete information on employment status during follow-up. We assumed that follow-up was synonymous with unemployment duration. In some studies this was clearly the case; in others, it was less so. This problem has been noted in another meta-analysis on unemployment duration in relation to all-cause mortality [7]. The high degree of heterogeneity between studies is another limitation in understanding the association between unemployment duration and suicide, which may be attributable to factors such as differences in how unemployment was measured (e.g., continuous [19]–[21]], ordinal [15], [24], or interval measurement [8], [14], [16], [17]), differences in study design and, differences in study populations. Variation may also reflect the notable dissimilarity in follow-up and the lack of information about the relationship between unemployment duration and suicide between five and 12 years. Geographical context, labour market opportunities (e.g., unemployment rates) and other contextual influences across the samples included in meta-analysis may also affect results.

Another notable problem was that some studies reported only adjusted results while others only provided crude results. A sensitivity analysis using meta-regression indicated no significant differences depending on whether results adjusted for covariates such as mental illness, age and SES. However, this analysis was limited by small sample size and a heterogeneous sample. It is possible that unmeasured confounders such as age and SES could wholly explain the observed association if no effect of unemployment on suicide existed. Information on the relationship between SES and suicide from a recent published paper published (kindly provided by Lundin [32]), indicated that the relationship between unemployment and suicide reduced after controlling for lower SES. Similarly, after controlling for age, the relationship between unemployment and suicide reduced. However, neither of these confounders attenuated the relationship between suicide and unemployment toward the null. The data in this recent paper [32] was used in several other studies included in the meta-analysis and therefore the results of this paper are likely to be generalizable to the present meta-analytic study.

Studies in the meta-analysis with over 12 years of follow-up also controlled for mental illness, which some researchers would argue explains a substantial amount of the relationship between unemployment and suicide [31]. This would suggest that cases of suicide among unemployed populations are a reflection of vulnerabilities that precede the loss of a job (i.e., the health selection hypothesis or the latent sickness hypothesis) [7], [15]. The smaller relative risk estimates in those studies with over five years of follow-up may indicate survivor bias, in that those participants remaining at longer periods of follow-up are less likely to be experiencing an ongoing mental illness [14], [15]. However, mental distress following unemployment has also been considered as a mediator of the relationship between unemployment and suicide, rather than a confounder [6]. Thus, controlling for mental illness is likely to lead to an underestimation of the relationship between unemployment and suicide. Similarly, adjusting for other likely intermediatories such as alcohol use and low emotional control would have the effect of biasing results towards the null. Other limitations include the possibility that a number of relevant articles were excluded in the review process. The review also excluded qualitative studies and case reports, articles not in English, and studies that were not published in the peer-reviewed literature.

In conclusion, the general finding of this review is that the long-term unemployed have a greater risk of suicide and attempted suicide compared to those unemployed in the short term, or compared to the general employed population. In order to better characterise the relationship between unemployment duration and suicide, there is a need for future studies to indicate a continuous measurement of employment status throughout the duration of follow-up. Future studies also need to capture individual influences on the relationship between unemployment duration and suicide, such as whether the individual has previously been unemployed, their occupation, and their age and sex, [7], [9]. Knowledge in this area would also benefit from an assessment of contextual factors affecting outcomes for long term unemployed such as the availability of jobs at a population level and social welfare programs for the unemployed [7], [9].

## Supporting Information

Table S1 **Papers assessing the relationship between unemployment and suicide.**
(DOCX)

Figure S1 **Funnel plot to assess publishing bias and small study effects, meta-analysis of suicide risk following unemployment, follow up over time.**
(TIF)

## References

[1] Jin RL, Shah CP, Svoboda TJ (1995) The impact of unemployment on health: a review of the evidence. CMAJ 153: 529. 7641151 PMC1487417

[2] Platt S (1984) Unemployment and suicidal behaviour: a review of the literature. Soc Sci Med 19: 93–115. 6382623 10.1016/0277-9536(84)90276-4

[3] Wilson SH, Walker GM (1993) Unemployment and health: a review. Public Health 107: 153–162. 8511234 10.1016/s0033-3506(05)80436-6

[4] Stack S (2000) Suicide: A 15-Year Review of the Sociological Literature Part I: Cultural and Economic Factors. Suicide Life Threat Behav 30: 145–162. 10888055

[5] Qin P, Agerbo E, Mortensen P (2005) Factors contributing to suicide: the epidemiological evidence from large-scale registers. Prevention and treatment of suicidal behavior: From science to practice. Oxford: Oxford University Press. pp. 11–28.

[6] Li Z, Page A, Martin G, Taylor R (2011) Attributable risk of psychiatric and socio-economic factors for suicide from individual-level, population-based studies: a systematic review. Soc Sci Med 72: 608–616. 21211874 10.1016/j.socscimed.2010.11.008

[7] Roelfs DJ, Shor E, Davidson KW, Schwartz JE (2011) Losing life and livelihood: a systematic review and meta-analysis of unemployment and all-cause mortality. Soc Sci Med 72: 840–854. 21330027 10.1016/j.socscimed.2011.01.005 PMC3070776

[8] Garcy AM, Vagero D (2012) The length of unemployment predicts mortality, differently in men and women, and by cause of death: A six year mortality follow-up of the Swedish 1992–1996 recession. Soc Sci Med 74: 1911–1920. 22465382 10.1016/j.socscimed.2012.01.034

[9] Paul KI, Moser K (2009) Unemployment impairs mental health: Meta-analyses.. J Vocat Behav 74: 264–282.

[10] StataCorp (2012) Stata Release 12.1. College Station, Texas StataCorp LP

[11] Harbord RM, Harris RJ, Sterne JAC (2009) Updated tests for small-study effects in meta-analyses. Stata Journal 9: 197–210.

[12] Sanderson S, Tatt ID, Higgins JP (2007) Tools for assessing quality and susceptibility to bias in observational studies in epidemiology: a systematic review and annotated bibliography. Int J Epidemiol 36: 666–676. 17470488 10.1093/ije/dym018

[13] Agerbo E (2005) Effect of psychiatric illness and labour market status on suicide: a healthy worker effect? J Epidemiol Community Health 59: 598–602. 15965145 10.1136/jech.2004.025288 PMC1757077

[14] Eliason M, Storrie D (2009) Does Job Loss Shorten Life? J Hum Resour 44: 277.

[15] Lundin A, Lundberg I, Hallsten L, Ottosson J, Hemmingsson T (2010) Unemployment and mortality—a longitudinal prospective study on selection and causation in 49321 Swedish middle-aged men. J Epidemiol Community Health 64: 22–28. 19289388 10.1136/jech.2008.079269

[16] Maki N, Martikainen P (2010) A register-based study on excess suicide mortality among unemployed men and women during different levels of unemployment in Finland. J Epidemiol Community Health 66: 302–307. 20966447 10.1136/jech.2009.105908

[17] Qin P, Agerbo E, Mortensen PB (2003) Suicide risk in relatiofindn to socioeconomic, demographic, psychiatric, and familial factors: a national register-based study of all suicides in Denmark, 1981–1997. Am J Psychiatry 160: 765–772. 12668367 10.1176/appi.ajp.160.4.765

[18] Mortensen PB, Agerbo E, Erikson T, Qin P, Westergaard-Nielsen N (2000) Psychiatric illness and risk factors for suicide in Denmark. The Lancet 355: 9–12. 10.1016/s0140-6736(99)06376-x 10615884

[19] Shah A (2008) Possible relationship of elderly suicide rates with unemployment in society: A cross-national study. Psychol Rep 102: 398–400. 18567209 10.2466/pr0.102.2.398-400

[20] Stack S, Haas A (1984) The Effect of Unemployment Duration on National Suicide Rates - a Time-Series Analysis, 1948–1982. Sociol Focus 17: 17–29. 12339403 10.1080/00380237.1984.10570459

[21] Classen TJ, Dunn RA (2012) The effect of job loss and unemployment duration on suicide risk in the United States: a new look using mass-layoffs and unemployment duration. Health Econ 21: 338–350. 21322087 10.1002/hec.1719 PMC3423193

[22] Platt S, Kreitman N (1990) Long term trends in parasuicide and unemployment in Edinburgh, 1968–87. Soc Psychiatry Psychiatr Epidemiol 25: 56–61. 2305313 10.1007/BF00789071

[23] Platt S, Kreitman N (1985) Parasuicide and unemployment among men in Edinburgh 1968–82. Psychol Med 15: 113–123. 3873081 10.1017/s0033291700020973

[24] Standish-Barry HM, Clayden A, Sims AC (1989) Age, unemployment and parasuicide in Leeds. Int J Soc Psychiatry 35: 303–312. 2628373 10.1177/002076408903500402

[25] Morton MJ (1993) Prediction of Repetition of Parasuicide: With Special Reference to Unemployment. Int J Soc Psychiatry 39: 87–99. 8340216 10.1177/002076409303900202

[26] Fergusson DM, Horwood LJ, Woodward LJ (2001) Unemployment and psychosocial adjustment in young adults: causation or selection? Soc Sci Med 53: 305–320. 11439815 10.1016/s0277-9536(00)00344-0

[27] Fergusson DM, Boden JM, Horwood LJ (2007) Unemployment and suicidal behavior in a New Zealand birth cohort: a fixed effects regression analysis. Crisis 28: 95–101. 17722691 10.1027/0227-5910.28.2.95

[28] Greenland S (1998) Meta-analysis. In: Rothman KJ, Greenland S, editors. Modern epidemiology. 2nd ed. ed. Philadelphia, PA: Lippincott-Raven. pp. 643–673

[29] Frese M, Zapf D (1988) Methodological issues in the study of work stress: Objective vs subjective measurement of work stress and the question of longitudinal studies. In: Cooper CL, Payne R, editors. Causes coping and consequences of stress at work Oxford, England: John Wily and Sons Lt. pp. 375–411.

[30] Kulik L (2001) Impact of length of unemployment and age on jobless men and women: a comparative analysis. J Employ Couns 38: 15–27.

[31] Blakely TA, Collings SC, Atkinson J (2003) Unemployment and suicide. Evidence for a causal association? J Epidemiol Community Health 57: 594–600. 12883065 10.1136/jech.57.8.594 PMC1732539

[32] Lundin A, Lundberg I, Allebeck P, Hemmingsson T (2012) Unemployment and suicide in the Stolkholm population: A register-based study on 771,068 men and women. Public Health 126: 371–377. 22480712 10.1016/j.puhe.2012.01.020

