# Supplementary material for: Long-Term Unemployment and Suicide: A Systematic Review and Meta-Analysis
Source: PLoS One. 2013 Jan 16;8(1):e51333. doi: 10.1371/journal.pone.0051333 (PMC3547020; doi:10.1371/journal.pone.0051333)
Supplement: Table S1 — Papers assessing the relationship between unemployment and suicide. (DOCX) [file pone.0051333.s001.docx]

Table S1. Papers assessing the relationship between unemployment and suicide.

| Author and year | Design | Population | Location and time period | Outcome | Age groups and sex | Measurement of unemployment duration | Analysis | Findings |
| --- | --- | --- | --- | --- | --- | --- | --- | --- |
| Agerbo (2005) [11] | Nested case-control | Labour market affiliation the two previous years among persons  admitted to a psychiatric hospital | Denmark | Suicide deaths | 25-60 years, males and females | 3 categories: Recent unemployment, 2 year history of unemployment compared, those employed. | Cox proportional hazards models, Logistic regression | Becoming unemployed (unemployed the  previous year, fully employed two  years earlier) OR 1.38 suicide (1.15 -1.65), Unemployed the two previous years before admission OR 1.01 (0.89-1.15) (after adjustment for psychiatric factors) |
| Classen & Dunn (2012) [19] | Ecological longitudinal study | Population - level | USA states, 1996 to 2005 | Suicide deaths | Suicide rates (ages 20-59 years), males only | Unemployment duration (12 month moving average, 1000s of workers) >5 wks, 5-14 wks, 15-26 wks, more than 26 wks. Measured as per 1000 workers. | Propensity score weighted estimation | >5 wks - male and female suicide15–26 wks + male and female suicide |
| Eliason & Storrie (2007) [12] | Cohort study | Population - level | Sweden | Suicide deaths | 25-64 years, males and females | Estimates of death at < 4 years, 5-8yrs, or 9-12 yrs after job loss from 760 establishment closures. | Propensity score weighted estimator | < 4 years HR 2.15 (CI 1.28-3.59) male suicide, non-sig 5-8 and 9-12 years later. Non-sig female suicide. Reference group was non-displaced workers. |
| Fergusson et al., (2007) [25] | Cohort study | Population – level, 1983 to 1999 | New Zealand | Suicide attempts | 16-25 years, males and females | Unemployed >6 months, unemployed 6 months +. Reference category is never unemployed in the previous year. | logistic regression | 6 months + suicidal ideation OR 1.43 (CI 0.96- 2.16) + suicide attempts OR 1.72 (CI 0.89 -3.32) (after adjustment for psychiatric factors) |
| Fergusson et al., (2001) [24] | Cohort study | Population – level, children born in 1977 | NZ | Suicide attempts | 16-21 years, males and females | Unemployed >3 months, unemployed 3-5 months, unemployed 6 months +. Reference category is never unemployed in the year. | logistic and Poisson  regression model | Those not unemployed had a suicide attempt rate of 1.4 (per 100 persons annually), those who had been exposed to 6 months or more of unemployment had a suicide attempt rate of 6.7 (per 100 persons annually). Controls were employed living population. |
| Garcy & Vagero (2012) [8] | Cohort study | Population - level, cohort born 1931 to 1965 | Sweden | Suicide deaths | 31 to 64 years | Interval level measure of accumulated measure of unemployment. | Cox proportional hazards models | Increased unemployment length + male suicide (HR 1.51, CI 1.38 – 1.66). No relationship apparent for female suicide (after adjustment for psychiatric factors). |
| Lundin et al., (2010) [13] | Cohort study | Population – level, born 1949-1950 | Sweden | Suicide deaths | 44-54 years, males only | Unemployment between 1 and 89 days, and over 90 days or more. Follow-up at 4 and 8 years. | cox proportional hazards models | During the first 4 years of follow up, >89 days + suicide HR 1.76 (0.89 -3.50). At 8 years follow up + suicide (HR 1.02, CI 0.42- 2.53) (after adjustment for psychiatric factors). Reference was employed living population. |
| Maki & Martikainen (2010) [14] | Cohort study | Population – level, | Finland | Suicide deaths | 25-64 years, males and females | Long term unemployed, those who had experienced some unemployment. Reference category was stably employed. Follow- up at 12 months. | Cox proportional hazards models | Compared to stably employed population, long term unemployed + male suicide (HR 2.72, CI 2.48-2.99), + female suicide (HR 3.27, CI 2.67-3.99) (after adjustment for psychiatric factors). Controls were employed living population. |
| Mortensen et al., (2000) [16] | Nested case-control | Population - level | Denmark | Suicide deaths | 16-78 years, males and females | Proportion of weeks in a year unemployment benefits were paid: <20%, 20-100%. Compared to working population | Cox proportional hazards model | Unemployment 20-100%  IRR 1.35 (1.03–1.76) (after adjustment for psychiatric factors). Matched to employed living population. |
| Morton (1993) [23] | Clinical cohort, 1984-1986 | Hospital | Scotland | Suicide attempts (repeater versus non-repeater) | 16-64 years, males only | Unemployment for up to 1 year versus over 1 year. | Relative risks | Repetition rate for suicide attempt 1985: 11.5% in those unemployed < 52 weeks, 21.1% in those unemployed > 52 weeks. In 1986, 8.6% in those unemployed < 52 weeks, 27.9% in those unemployed > 52 weeks. RR 1.8 in 1985 and 2.5 in 1986. |
| Platt & Kreitman (1985) [21] | Cohort study based on hospital admissions, chi-square, relative risks | Hospital | UK | Suicide attempts | Suicide rate (ages 15 and over), males | Unemployment < 4 weeks, 5-26 weeks, 27-52 weeks, > 52 weeks. Reference category was employed. | Relative risks | RR < 4 weeks 8.8, RR 5-26 weeks 5.4, RR 27-52 weeks 10.4, the RR > 52 weeks was 18.9. |
| Platt & Kreitman (1990) [20] | Cohort study based on hospital admissions, chi-square, relative risks | Hospital | UK | Suicide attempts | Suicide rate (ages 15 and over), males and females | Unemployment < 4 wks, between 5-26 weeks, 27-52 wks, >52 weeks. Reference category was employed workforce. | Mortality rates | Unemployed >52 weeks RR between 2 - 5 than those unemployed < 4 weeks. |
| Qin et al., (2003) [15] | Cohort study 1991-1992 | Population-level | Denmark | Suicide deaths | 9-103 years, males and females | Proportion of weeks in a year unemployment benefits were paid: <20%, 20-80%, >80% | Logistic regression | Unemployment <20%  OR 1.11 (1.03-1.20), 20-80% OR1.18 (1.10-1.27), 81-100% OR 1.21 (1.07-1.36) male suicide. Unemployment <20%  OR 1.23 (1.06-1.43), 20-80% OR1.15 (1.01-1.31), 81-100% OR 1.19 (0.97-1.46) female suicide (after adjustment for psychiatric factors). Matched living controls. |
| Shah (2008) [17] | Ecological longitudinal study | Population-level | 27 OECD countries | Suicide deaths (alcoholics) | Suicide rate (ages 65 year and over), males and females | Long term unemployment, time unspecified. Measured as a continuous variable. | Correlation | No relationship between elderly suicide rates (65-74, 75+) and long term unemployment. |
| Stack & Haas (1984) [18] | Ecological longitudinal study | Population-level | USA | Suicide deaths (alcoholics) | All age suicide rates, males and females | Average duration. Measured as a continuous variable. | Cochrane-Orcutt iterative procedure | Duration of unemployment  + male and female suicide rates (after adjustment). |
| Standish-Barry et al., (1989) [22] | Hospital-based, 1978-1982 | Population | UK | Suicide attempts | Under 20-59 years, males and females | Those unemployed < 52 weeks, compared unemployment >52 weeks. | Correlation | Long term unemployment (> 52 weeks) + male parasuicide rates 50-54 years (0.59, p<0.01), -55-59 years (-0.69, p<0.01). |

** OR= odds ratio; RR= Relative risk; IRR= incidence rate ratio; HZ= hazard ratio; CI=95% confidence intervals; + positive relationship, - negative relationship
