# Supplementary figures and images for: Long-Term Unemployment and Suicide: A Systematic Review and Meta-Analysis
Source: PLoS One. 2013 Jan 16;8(1):e51333. doi: 10.1371/journal.pone.0051333 (PMC3547020; doi:10.1371/journal.pone.0051333)

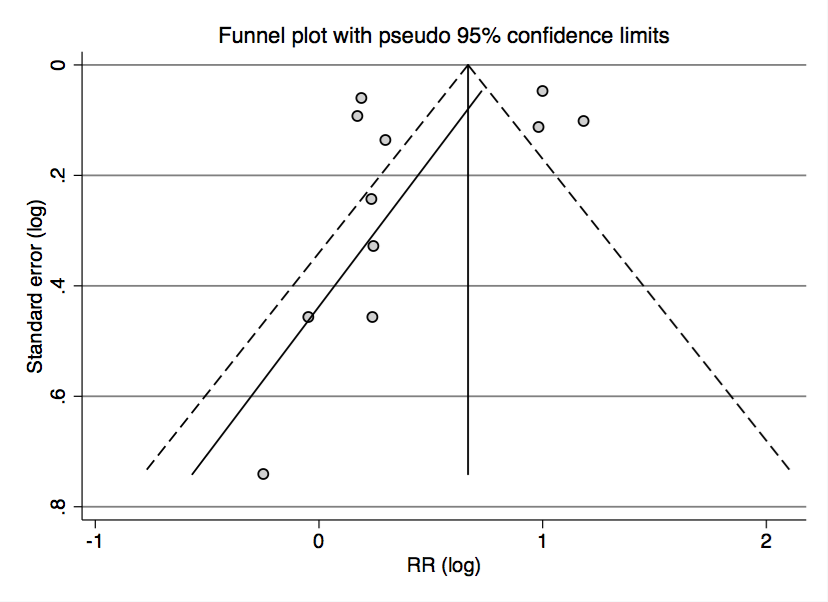

Supplement: Figure S1 — Funnel plot to assess publishing bias and small study effects, meta-analysis of suicide risk following unemployment, follow up over time. (TIF) [file pone.0051333.s002.tif]
